# Gamma radiation assisted green synthesis of hesperidin-reduced graphene oxide nanocomposite targeted JNK/SMAD4/MMP2 signaling pathway

**DOI:** 10.1038/s41598-024-60347-5

**Published:** 2024-05-21

**Authors:** Ahmad S. Kodous, Eman. O. Taha, Dina F. El-Maghraby, Asmaa A. Hassana, M. M. Atta

**Affiliations:** 1 Radiation Biology Department, National Center for Radiation Research and Technology (NCRRT), Egyptian Atomic Energy Authority (EAEA), Cairo, Egypt; 2 Petroleum Applications Department, Egyptian Petroleum Research Institute (EPRI), Cairo, Egypt; 3 Health Radiation Research Department, National Center for Radiation Research and Technology (NCRRT), Egyptian Atomic Energy Authority (EAEA), Cairo, Egypt; 4 Radiation Physics Department, National Center for Radiation Research and Technology (NCRRT), Egyptian Atomic Energy Authority (EAEA), Cairo, Egypt

**Keywords:** Hesperidin, Graphene, Radiation, HepG2, Anti-metastasis, JNK/SMAD4/MMP2, Biochemistry, Chemical biology, Chemistry, Materials science

## Abstract

In this study, a novel method for the fabrication of hesperidin/reduced graphene oxide nanocomposite (RGOH) with the assistance of gamma rays is reported. The different RGOHs were obtained by varying hesperidin concentrations (25, 50, 100, and 200 wt.%) in graphene oxide (GO) solution. Hesperidin concentrations (25, 50, 100, and 200 wt.%) in graphene oxide (GO) were varied to produce the various RGOHs. Upon irradiation with 80 kGy from γ-Ray, the successful reduction of GO occurred in the presence of hesperidin. The reduction process was confirmed by different characterization techniques such as FTIR, XRD, HRTEM, and Raman Spectroscopy. A cytotoxicity study using the MTT method was performed to evaluate the cytotoxic-anticancer effects of arbitrary RGOH on Wi38, CaCo2, and HepG2 cell lines. The assessment of RGOH’s anti-inflammatory activity, including the monitoring of IL-1B and IL-6 activities as well as NF-kB gene expression was done. In addition, the anti-invasive and antimetastatic properties of RGOH, ICAM, and VCAM were assessed. Additionally, the expression of the MMP2-9 gene was quantified. The assessment of apoptotic activity was conducted by the detection of gene expressions related to BCl2 and P53. The documentation of the JNK/SMAD4/MMP2 signaling pathway was ultimately accomplished. The findings of our study indicate that RGOH therapy has significant inhibitory effects on the JNK/SMAD4/MMP2 pathway. This suggests that it could be a potential therapeutic option for cancer.

## Introduction

Cancer follows cardiovascular diseases as the second leading cause of death worldwide^1^. Owing to the constantly growing universal occurrence of malignancies, innovative, efficient therapeutics and treatment approaches are required^1^. Hepatocellular carcinoma (HCC) is a prevalent type of cancer in Egypt and worldwide, which is responsible for many cancer-related deaths due to its aggressiveness and poor diagnosis^2^. As the prevalence of malignancies is increasing globally, there is a need for more effective and innovative treatment approaches^1^. Safe and naturally occurring substances with potent anticancer characteristics, such as fruits, grapes, and vegetables, can provide novel possibilities in cancer therapy^3^. Citrus fruits like lemons and oranges are well recognized for their chemopreventive properties. Hesperidin, which is also known as hesperetin 7-rutinoside, is a flavonoid compound with considerable importance as a citrus bioactive constituent^1^.

The absence of a double bond between positions 2 and 3 in the C ring of flavanones distinguishes them from other well-known types of flavonoids, such as flavones, flavonols, or isoflavones. Hesperidin has been shown to possess various pharmacological properties, demonstrating significant efficacy as an anti-atherosclerotic, antioxidant, cardioprotective, anti-inflammatory, anti-diabetic, anti-allergic, neuroprotective, antimicrobial, and anticancer agent^4–7^.

Graphene with a 2D honeycomb structure has stunning features such as optimum transparency and admirable electronic and mechanical properties^8,9^. Scientists studied the properties of graphene-based materials, and they found that it is an excellent material for many applications such as bio-sensing^10^, supercapacitors^11^, hydrogen production^12^, ferromagnetic tunneling diodes^13^, molecular drug delivery^14^, polymer nanocomposites^15,16^, cancer treatment^17^, and bone tissue engineering^18^. Also, the development of graphene nanomaterials has led to advancements in integrated cancer diagnosis and therapy. These nanomaterials have other bioactive molecules, such as chemotherapeutic agents, nano-polymers, antibodies, and more^19,20^.

Reduced graphene oxide (RGO) is one of the graphene derivatives which shows graphene-like properties with a low cost of production^21^. Synthesis of RGO implies the reduction of graphene oxide (GO) via different reported approaches such as chemical^22,23^, electrochemical^24^, microwave^25^, and thermal^26^. In addition, γ-rays were used as a convenient, cost-effective, and less harsh method for synthesizing RGO^9,27,28^. Due to their lack of toxicity, simplicity, and environmental friendliness, several green reducers, such as planet extract, bacteria, amino acids, and organic acids, are also employed for the green reduction of GO^29^.

The current study aims to synthesize biomaterials based on RGO functionalized with different Hesperidin ratios with the assistance of γ-rays as an eco-friendly, one-step, and facile approach. Different techniques are employed to study and characterize the prepared samples. The study also aims to evaluate the cytotoxicity and anticancer effects of the prepared biomaterials on normal fibroblast (Wi38), colorectal (CaCo2), and hepatoma (HepG2) cancer cell lines. The current data postulated that RGOH100 has antimetastatic and anticancer activity against the HepG2 cell line by inducing apoptosis, inhibiting adhesion molecules, and prohibiting the c-Jun N-terminal kinase, main signal transducer for receptors of transforming growth factor-β (TGF-β), and matrix metalloproteinase-2 (JNK/SMAD4/MMP2) pathway.

## Materials and methods

### Materials

Graphite (particles less than 50 µm) was obtained from Merk, a company based in Germany. The following chemicals were acquired from El Nasr Pharmaceutical Chemicals Company in Egypt: hydrogen peroxide (H_2_O_2_, 35%), phosphoric acid (H_3_PO_4_, 85%), potassium permanganate (KMnO_4_), sulfuric acid (H_2_SO_4_, 98%), ethanol (96% concentration), and hydrochloric acid (HCl). The compound hesperidin was acquired from BDH Chemicals Ltd., an England-based company. The MTT reagent used in this study was 3-(4,5-dimethylthiazol-2-yl)-2,5-diphenyltetrazolium bromide (Sigma, Germany). The HepG2, CaCO2, and Wi8 cell lines were obtained from the tissue culture division of VACSERA, Egypt, a prominent organization specializing in biological products and vaccines.

### Production of graphene oxide

Fabrication of GO was conducted via the improved Hummer process, as described earlier^30^. Briefly, one gram of graphite was dissolved in an ice bath containing a 3:1 solution of H_2_SO_4_ and H_3_PO_4_. Approximately 24 h were spent stirring the mixture continuously while 6 g of KMnO_4_ were added slowly. The volume of the suspension was increased to 300 ml through additional deionized water additions. The color of the mixture changed from deep purple-green to a rich brown. To prevent further oxidation, 30 mL of H_2_O_2_ solution was subsequently introduced. After washing the produced GO solid with a 1 M HCl aqueous solution, the solid was cleansed with deionized water until the pH reached 3^11^.

### Gamma irradiation source

The irradiation process was conducted using a system of γ-irradiation, which used a ^60^Co source for radiation. The dose rate during irradiation was around 0.9 kGy per hour, and the irradiation dose was 80 kGy.

### Synthesis of hesperidin reduced graphene oxide (RGOH) nanocomposites by γ-ray

Four samples, consisting of 100 mg of GO, were solubilized in 100 mL of distilled water. and exposed to ultrasonication for ~ 30 min. The hesperidin powder was introduced into the abovementioned combination at concentrations of 25, 50, 100, and 200 wt% relative to GO. Subsequently, the mixture underwent ultrasonication for 5 min.

Following the irradiation process, the resultant sample powder underwent a washing procedure using distilled water and then a drying process at 50 °C for about 6 h. The samples were categorized based on their hesperidin concentration, specifically RGOH25, RGOH50, RGOH100, and RGOH200.

## Characterization techniques

The produced samples underwent structural investigation using Shimadzu X-ray diffractometer with CuK radiation (λ = 1.5405A°). All measurements were conducted using the continuous scan mode, covering a scan range of 4–90°. The voltage across the generator was measured to be 40 kV, while the current flowing through the generator was recorded as 30 mA. The chemical compositions of RGOHs were analyzed via FT-IR; Shimadzu Prestige-21, operating within frequency range of 4000–500 cm^−1^. The structural flaws in the materials were evaluated at room temperature using Witec Alpha 300 R confocal Raman spectroscopy equipped with aNd: Yag laser excitation source at a wavelength of 532 nm. The spectral range analyzed was between 1000 and 2000 cm^−1^. The materials' surface morphology was investigated by High-Resolution Transmission Electron Microscope (a JEOL-JEM2100) at operating voltage of 200 kV^31,32^.

### In vitro cytotoxicity assay (MTT assay)

The HepG2 cancer, CaCo2 cancer, and Wi38 cells were cultured in high-glucose Dulbecco's Modified Eagle's Medium (H-DMEM) containing 10% fetal bovine serum (FBS), and 1% penicillin–streptomycin solution (50 unit/mLPenicillin, 50 µg/mL Streptomycin, and 0.25 µg/mL Amphotericin-B) and 13.5 g/L sodium bicarbonate at 37 °C in a humidified environment with 5% CO2. Prior to the experiment, the cells were grown until confluence was attained.

A MTT in vitro assay was used to measure the RGOHs cytotoxicity according to Burton (2005) with modifications. MTT reagent (3-(4,5-dimetylthiazol-2-yl)-2, 5-diphenyltetrazolium bromide) (Sigma, Germany) was performed on Wi38, HepG2, and CaCo2 cell lines. (100% viable) HepG2, CaCo2, and Wi38 cells in the media without treatment were considered controls.

RGOH100 was added complete monolayer sheet at concentrations ranging from 0 to 1600 µg/mL. After a full day (24h), the supernatants were removed, and the cell layers were subjected to a wash using phosphate buffered saline (PBS, Invitrogen Gibco). Subsequently, the cell layers were incubated with a 300 µl solution of MTT/well (0.5 mg MTT/mL) inside a 5% CO_2_ incubator for 4 h. Following that, the cells underwent centrifugation at a magnitude of 15,000 times the acceleration owing to gravity (15,000 × *g*) for 5 min. A volume of 200 μl of dimethyl sulfoxide (DMSO) was introduced subsequent to the removal of the culture medium. Following that, the samples were aggressively vortexed, and the optical density (O.D.) was determined at a wavelength of 560 nm. The absorbance of cells that were not subjected to any treatment was designated as 100%. Each sample and control were analyzed in triplicate in three separate tests. The percentage growth (%) of viable cells that were subjected to various treatments was determined using the following calculation method^33–37^:

$$\% {\text{ viable}}\, = \,{\text{sample abs}}/{\text{control abs}}\, \times \,{1}00.$$

### Biochemical assays

The biochemical markers were detected before and after incubation 33.29 µg/mL RGOH100 with HepG2 cancer cell line for 24 h. The IL6 concentration was determined with the use of an ELISA kit provided by Quantikin R&D System Catalog: D6050, USA. Using an enzyme-linked immunosorbent assay (ELISA) kit from MyBiosource (MBS701896) and following the instructions given by the vendor, the amount of IL1β was measured. The ICAM ELISA Kit (CD50) (ab275097) is a commercially available enzyme-linked immunosorbent assay (ELISA) kit. VCAM1 ELISA Kit (ab223591).

### Real-time polymerase chain reaction (RT-PCR)

The genes expression was detected before and after incubation 33.29 µg/mL RGOH100 with HepG2 cancer cell line for 24 h. The RNA was isolated from the cell line via the RNeasy Mini Kit in accordance with the strategies provided by the manufacturer. The complementary DNA (cDNA) was generated using the extracted RNA, which ranged from 0.5 to 2 µg. Real-time PCR (Promega, Madison, WI) was used to measure the amounts of tumor suppressor (P53), B-cell lymphoma 2 (Bcl2), MMP2, MMP9, SMAD4, JNK, and nuclear factor kappa-light-chain-enhancer of activated B cells (NF-kB) genes. Specific primers for each gene, together with GAPDH as a housekeeping gene (Table 1), were used in this analysis. The data were subjected to analysis using GraphPad Prism v 8.0 (GraphPad Software, La Jolla, CA, USA, https://www.graphpad.com/). Quantification was made via the v1.7 Sequence Detection Software. By using the comparative threshold cycle approach, it was possible to determine the relative expression of each target gene. The analysis included examining the variations in mRNA target content concerning the glyceraldehyde-3-phosphate dehydrogenase gene, which was normalized. This analysis was conducted using the comparative cycle threshold approach designated by Livak and Schmittgen^38–41^.

**Table 1:** Primer sequences employed for RT-PCR Primer Sequence.

NF-_k_B	F: 5'- CTCCGCGGGCAGCATCC -3' R: 5'- AGCCGCACAGCATTCAGGTCGTAG -3'
Bcl-2	F:5′-CGGGAGAACAGGGTATGA -3′ R:5′-CAGGCTGGAAGGAGAAGAT-3′
MMP 2	F: 5′- CCGAGGACTATGACCGGGATAA-3′ R: 5′- CTTGTTGCCCAGGAAAGTGAAG-3′
MMP 9	F:5′- CAGGATAAACTGTATGGCTTCTGC -3′ R:5′- GCCGAGTTGCCCCCA -3′
SMAD 4	F: 5′-ACATTGGATGGGAGGCTTCA-3′ R: 5′-GATCAGGCCACCTCCAGAGA-3′
JNK	F: 5'-TCTGGTATGATCCTTCTGAAGCA-3' R: 5'-TCCTCCAAGTCCATAACTTCCTT-3'
P53	F: 5'-AGGCCTTGGAACTCAAGGAT-3′ R: 5'-TGAGTCAGGCCCTTCTGTCT -3′
GAPDH	F: 5'- CTCCCATTCTTCCACCTTTG-3′ R: 5′- CTTGCTCTCAGTATCCTTGC-3′

## Results and discussion

### Characterization of hesperidin reduced graphene oxide nanocomposites

**Figure 1:**
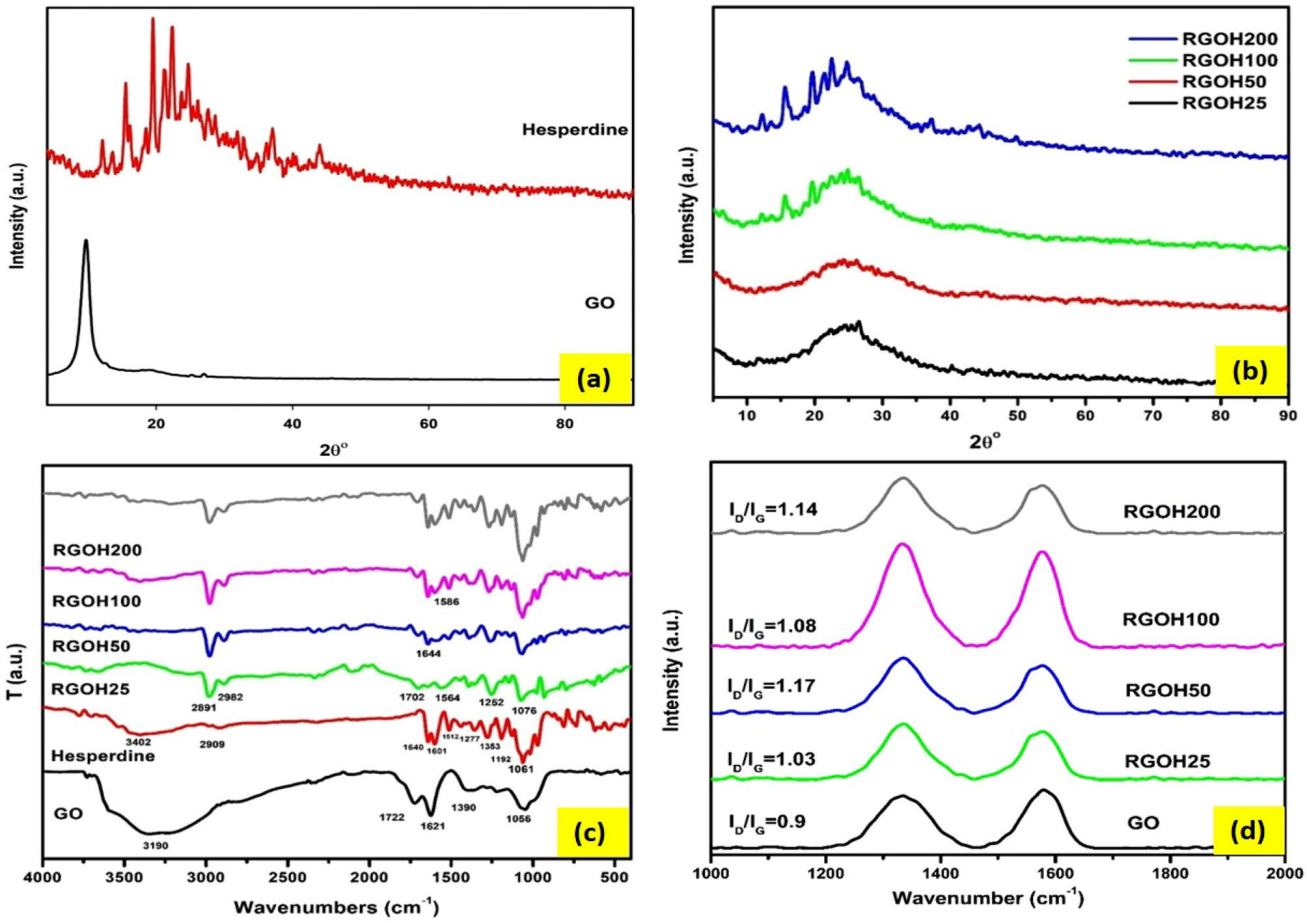
XRD of GO and pristine Hesperidin (**a**), XRD of different RGOH nanocomposites (**b**), FTIR of GO, Hesperidin, and RGOH nanocomposites (**c**), Raman spectra of GO and different RGOH nanocomposites (**d**).

Figure 1a demonstrates XRD for pristine GO and hesperidin. GO displays characteristic peaks at 9.6°, ascribed to the (001) reflection plane of GO sheets^42^. Pure hesperidin shows strong characteristic peaks at 8.5°, 12.1°, 15.5°, 19.5°, 22.3°, 23.7°, 24.6°, 27.6°, and 28.6° which demonstrates its high crystallinity^43,44^. Moreover, the XRD pattern of different RGOH nanocomposites is illustrated in Fig. 1b. The XRD of RGOH25 revealed the absence of a GO peak and a new broad peak at 24.5° was observed. These changes indicate that oxygen functional groups were efficiently removed from GO by hesperidin and γ-rays, and a successful reduction occurred^45^. There are no obvious hesperidin peaks in RGOH25 and RGOH50 due to interference from the RGO broad peak at low hesperidin concentrations. In contrast, hesperidin peaks became more prominent with increasing concentration, as shown in RGOH100 and RGOH200 patterns, indicating hesperidin maintains its crystalline structure^46^.

Figure 1c reveals the FTIR spectra of GO, hesperidin, and RGOH nanocomposites. The FTIR analysis of GO indicated the existence of many oxygen functional groups. This was evident from the strong and wide band detected at ~ 3200 cm^−1^, which endorsed the stretching vibrations of O–H bonds. The spectral peaks at 1722, 1621, and 1309 cm^−1^ designate the stretching vibrations associated with C=O, C=C, and C–OH, respectively. Furthermore, the researchers discovered the stretching vibrations of C-O at wavenumbers of 1230 cm^−1^ and 1056 cm^−1^^47,48^.

The FTIR spectra of Hesperidin demonstrate an absorption band at 3402 cm^−1^ owed to O–H stretching vibration. The bands at 2909 cm^−1^ ascribed to the C-H stretch of CH. The bands at 1640 cm^−1^ correspond to the C=O stretch, while those at 1601, 1512 and 1353 cm^−1^ ascribed to the C=C stretching of the aromatic group. Absorptions at 1277, 1192, 1127, and 1061 cm^−1^ related to the C-O stretch^43,44^. The observed drop in the intensity of oxygen functional bands associated with RGOH groups confirms the effective GO reduction^49^. Also, new peaks observed at 2982 and 2891 cm^−1^ suggest the formation of C-H groups upon the interaction of hesperidin and GO^3^.

Raman spectroscopy is an important and non-invasive technique used for the analysis of carbon-material structural properties. Figure 1d illustrates the Raman spectra of GO and RGOHs. Two distinct bands were seen in the GO spectra, namely the D band (situated at 1335 cm^−1^) and the G band (situated at 1580 cm^−1^). The G band is indicative of crystalline graphite in the E_2g_ zone center mode. The D band signifies the presence of disrupted symmetry at the edges of the flaws within the specimen^50^. Additionally, it can be seen that all samples of RGOH exhibit a greater widening of the D and G bands, which suggests a larger degree of disorder in these samples^50^.

The ID/IG ratio, which represents the proportion of D and G band intensities, is often used as an indicator of the level of structural flaws in graphitic materials^51^. Based on the data revealed in Fig. 1, the ID/IG values for all RGOH nanocomposites are higher than those of GO. This suggests a higher degree of defects and successful reduction, which is in line with findings reported in earlier studies^52^. Furthermore, it can be concluded that RGOH50 exhibits the greatest ID/IG ratio, indicating a greater prevalence of smaller graphitic domains in comparison to the other samples^53^.

**Figure 2:**
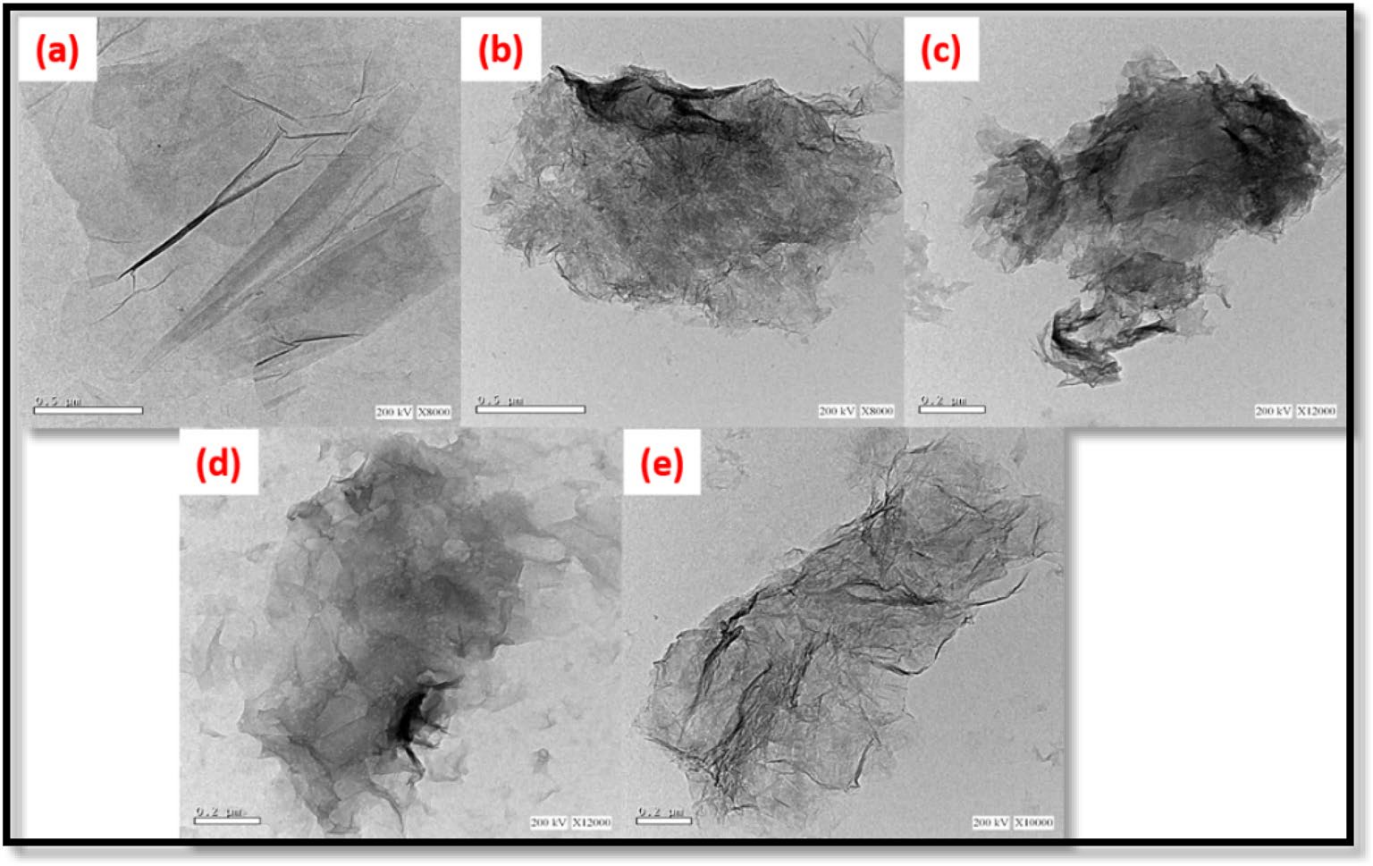
HRTEM images of GO (**a**), RGOH25 (**b**), RGOH50 (**c**), RGOH100 (**d**) and RGOH200 (**e**).

Figure 2 presents HRTEM images of GO and RGOHs. The HRTEM picture of GO reveals the presence of clear and pliable sheets with little wrinkling, indicating successful exfoliation into many layers. This observation aligns with the results documented in the literature^54^. The crumpled shape and folded look of the RGOH sheets characterize RGOH nanocomposites. These observations indicate the reduction process, which is consistent with findings described in the literature^45^.

### MTT in Vitro cytotoxicity assay

**Figure 3:**
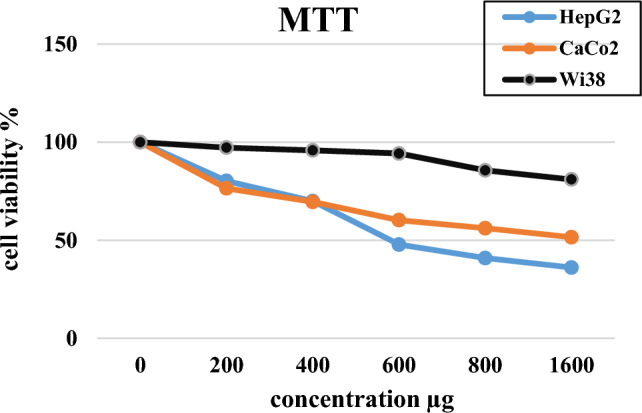
Cell viability of HepG2, CaCo_2_, and Wi38 cell lines incubated with different doses of RGOH100. (The values are displayed as mean ± SEM (n = 3) of two independent experiments.

The MTT test is a well-accepted in vitro model used for the assessment of the cytotoxicity of chemical compounds on cancer cell lines as well as for the screening of compounds that may possess anti-tumor characteristics^55^. Different doses of RGOH100 (as arbitrary samples among RGOHs) were studied on HepG2 (HCC cell line), CaCo2 (colon cancer cell line), and Wi38 (normal fibroblastic cell line). RGOH100 concentration ranges from 0 to 1600 µg/mL. The MTT data showed that increasing the RGOH100 doses decreased viability in both cancerous cell lines; HepG2 was 36.06% and CaCo2 was 51.53% relative to the controls (100%). However, the effect of RGOH100 on the normal fibroblast cell line Wi38 showed viability (97.2–81%) in concentrations 0–1600 µg, respectively, confirming the selective impact of RGOH100 against cancer cell lines, as seen in Fig. 3^56,57^. Furthermore, the IC_50_ results of RGOH100 documented that RGOH100 IC_50_ is non-toxic for normal cells (Wi38) (564.9 µg/mL), but it has a potent cytotoxic influence on the cancer cell lines CaCo2 (98.58 µg/mL) and HepG2 (33.29 µg/mL), which means it may have an anticancer potential against cancer cell lines.

The RGOH100 IC_50_ values in Wi38, CaCo2, and HepG2 cell lines were 564.9, 98.58, and 33.29 µg/mL, respectively.

### Biochemical investigations

**Figure 4:**
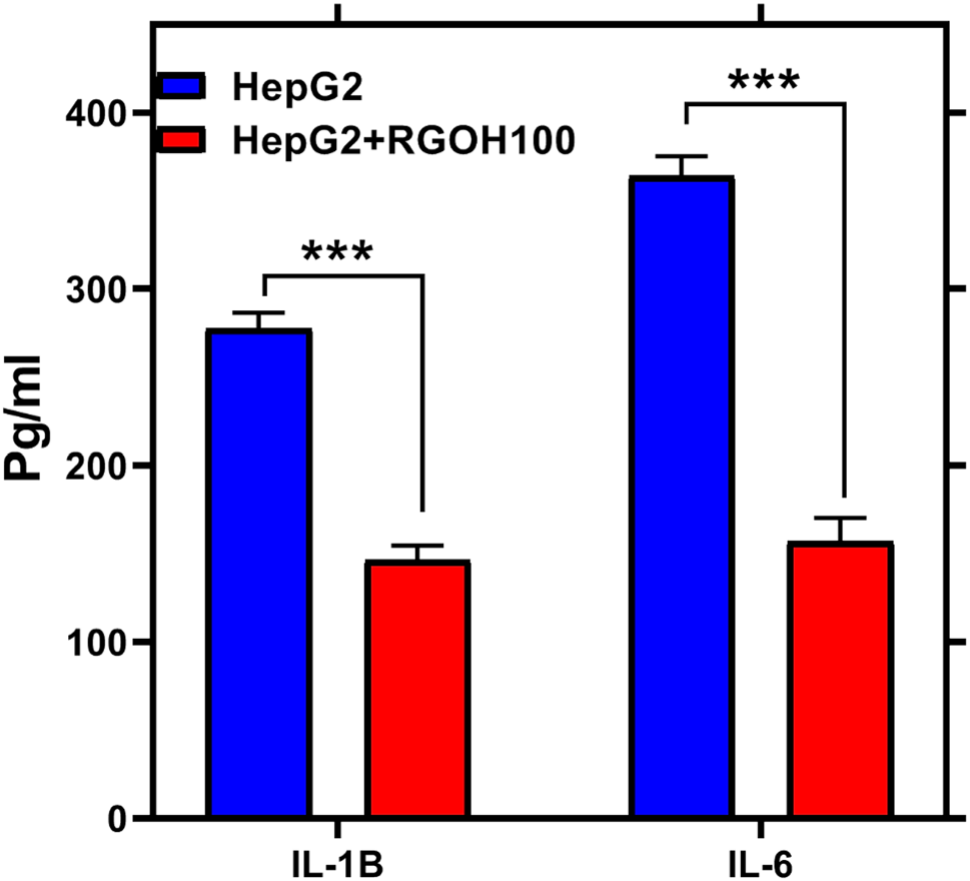
Effect of RGOH100 treatment in HepG2 cell line on the proinflammatory markers IL-1β (pg/mL) and IL-6 (pg/mL). Each value denotes the mean ± SE (n = 6). The mean values indicate statistically significant changes from the control group (HepG2), significance level of ***p < 0.001.

The pro-inflammatory markers IL-1β and IL-6 levels were examined and illustrated in Fig. 4. In the HepG2 (T) group, IL-1β was recorded at 277.6 ± 5.12 and IL-6 at 364.23 ± 6.4. However, after treatment with RGOH100, there was a marked decrease in both markers, with IL-1β at 146.5 ± 4.75 and IL6 163.3 ± 8.2. Kadhim and his colleagues^58^ found that RGO can decrease IL-6, and Hoyle et al.^59^ showed that RGO did not induce IL-6 at any dose. Moreover, the current data is in accordance with a prior investigation that revealed that RGOH100 can inhibit the production of pro-inflammatory cytokines such as IL-1β and IL-6^44,60^**.**

Tumor cells often cause immunological tolerance by secreting suppressive chemicals such as IL-10, TGF-b, prostaglandin E2, and VEGF^61^. Tumor cells also secrete IL-1, IL-4, IL-6, IL-10, and M-CSF to stimulate M2-like macrophage differentiation to limit immunological attack^62,63^. Macrophages, known as Kupffer cells in the liver, prevent early HCC formation. However, as the tumor progresses, M1 becomes M2, suppressing the immune system and supporting the tumor^64^. M2 macrophages increase tumor development and metastasis, and liver tumors with TAM infiltration have poor prognoses. In the current research, RGOH100 therapy reduced HepG2 production of immunosuppressive cytokines IL-1β and IL-6^65^. This inhibition prevented HepG2 cells from becoming M2-like macrophages, improving anti-tumor immunity.

**Figure 5:**
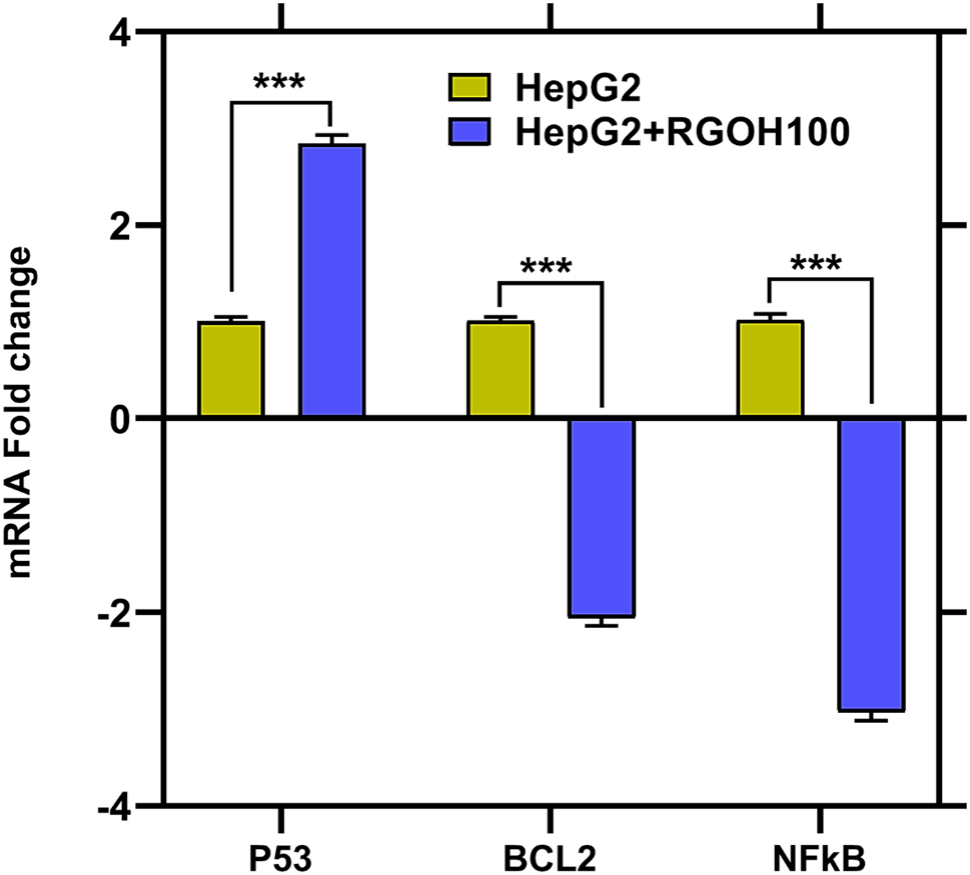
The gene expression of p53, BCl2, and NF-kB after RGOH100 treatment in cells HepG2. Every individual value denotes the mean ± SE (n = 3). The mean values indicate significant differences (***p < 0.001) when compared to HepG2 as the control.

The p53 protein is a nuclear transcription factor responsible for activating genes that control cell cycle arrest and apoptosis^66^. A study that looked at the expression of the p53 gene found that the group that was treated with RGOH100 had a much higher level of p53 gene expression (284%) than the HepG2 group. In comparison, the anti-apoptotic protein BCl2 was significantly reduced (-106%) compared to the HepG2 group (P < 0.05), as revealed in Fig. 5. These findings align with the work of Amaral et al.^67^, who have suggested that p53 triggers apoptosis by activating pro-apoptotic proteins bcl-2-like protein 4 (Bax and Bak) and suppressing anti-apoptotic genes such as BCl2 and surviving, thus activating the caspase pathway^68^.

By altering membrane- and cytoskeleton-associated gene expression, GO may damage cells. After internalization, GO may localize on F-actin filaments and cause cell cycle arrest, death, or oxidative stress^69^. ROS generation and antioxidant elimination imbalances cause oxidative stress. Cells create ROS during mitochondrial respiratory chain operation. ROS such as superoxide anion (O_2_–), hydrogen peroxide (H_2_O_2_), and hydroxyl radical (OH·) are crucial to cell metabolism. While ROS are pro-tumorigenic, excessive ROS levels are cytotoxic^70^. It was previously found that RGO generated intracellular ROS in MDA-MB-231 and ZR-75–1 breast cancer cell lines^71^. Increased ROS generation may damage mitochondria and alter ΔΨm in cancer cells^70^.

Apoptosis causes cell death via shrinking, membrane blebbing, DNA breakage, and apoptotic bodies. Two primary apoptotic routes are external (death receptor) and intrinsic (mitochondrial)^72^. Due to decreased mitochondrial membrane potential (MMP) and alterations in apoptotic cell shape, we believe RGO induces mitochondrial apoptosis in MDA-MB-231 and ZR-75–1 cells. Cancer cells' mitochondrial apoptosis mechanism depends on mitochondria. Incubating cells with GO disrupts the mitochondrial membrane, according to Lammel et al.^73^. This was linked to a reduction in MMP^74,75^.

Nanomaterials cause apoptosis by decreasing BCL-2 antiapoptotic protein expression and increasing BIM and BAX production. We found that rGO raised BAX and lowered BCL-xL and BCL-2. BIM enhances apoptosis by binding and neutralizing antiapoptotic proteins^76^. The α-helical domains of Bax protein aid in the development of mitochondrial membrane channels. BAX protein promotes mitochondrial membrane cytochrome c permeability via permeability transition holes. Apaf-1-cytochrome c complex stimulates procaspase-9. In response to various apoptotic triggers, caspase-9 plays a key role in the mitochondrial or intrinsic apoptotic pathway^76^. Active caspase 9 activates executive caspase-3^72^. PARP stimulates caspases-3 and -7 for apoptosis.

NF-κB is a transcription factor that plays a critical role in inflammation and cancer^77^. It induces the transcription of many pro-inflammatory cytokines, such as interleukins IL-6 and IL-8, secreted to boost the immune response. NF-κB is also activated in many cancers, which enhances the growth and survival of cancer cells, potentially explaining the increase found in the untreated HepG2 group. Moreover, the interplay between p53 and NF-κB plays an essential function in tumor development^78^. NF-κB is often up-regulated in tumor cells deficient in p53^79^. After the treatment of HepG2 cells with RGOH100, NF-κB declined (-203%) compared to the HepG2 group. These data are in harmony with Kretowski et al.^71,80^, who found that RGO decreased the NF-κB P65 subunit in breast cancer cells, which is related to apoptosis induction. Aggarwal et al.^1^ have suggested that hesperidin, especially in nanoform, induces p53 and attenuates NF-κB. Additionally**,** Ghorbani et al.^81^ have documented that hesperidin causes B cell precursor leukemia (NALM-6) cells to undergo apoptosis via inducing p53 and suppressing NF-κB activity.

**Figure 6:**
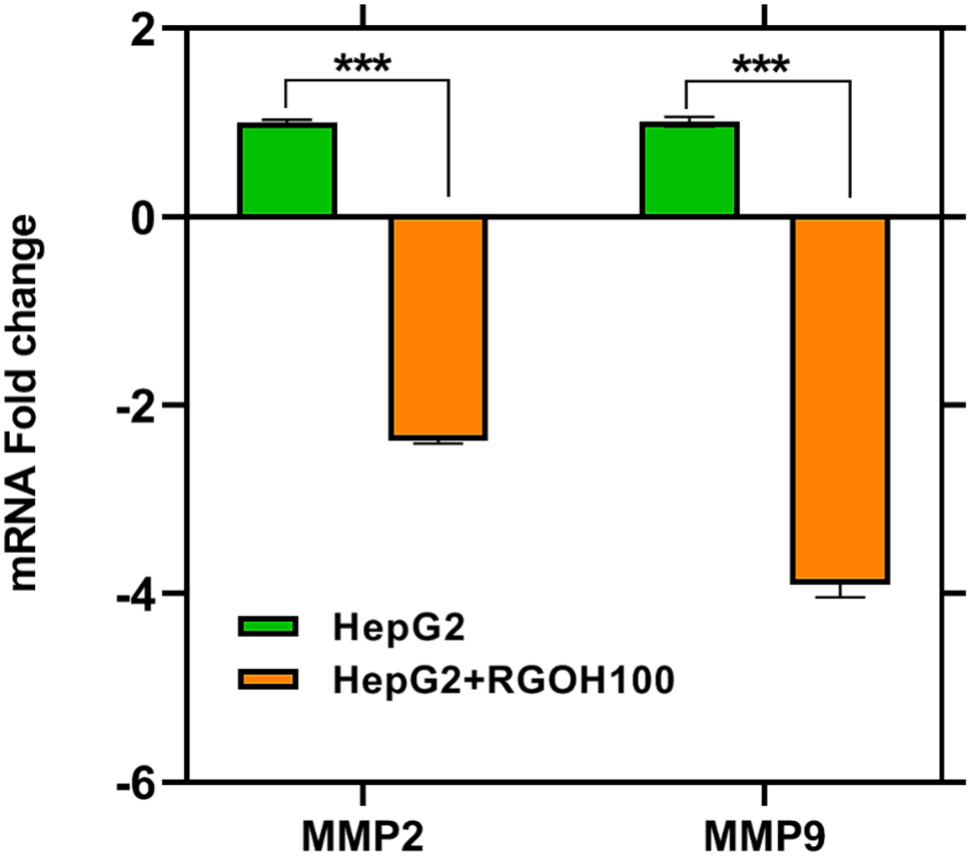
The gene expression of MMP2 and MMP9 as ΔCT and fold gene expression in HepG 2 cell line and treatment with RGOH100 (n = 3). The mean ± SE (n = 3). Mean values indicate statistically significant differences (***p < 0.001) when compared to HepG2 as the control.

Metastasis is a complex procedure involving the basement membrane's degradation, avoidance from the primary tumor, surviving in the circulatory system, colonizing, and increasing the targeted organ's parenchyma^82^. This research endeavored to examine the function of RGOH100 in the metastasis process by analyzing metastasis-related proteins such as matrix metalloproteinases (MMPs 2 and 9) and cell adhesion molecules (CAMs) (ICAM, VCAM). After treatment with RGOH100, both metalloproteases showed a significant decline, with a percent change recorded at − 137% and − 290% for MMP2 and MMP9, respectively, compared to the HepG2 group; as seen from Fig. 6. This agrees with the findings of Wang et al.^83^.

Xi et al.^84^ revealed the potential of RGO in cancer therapy, which has been revealed by its capability to induce apoptosis in cancer cells and impede their migratory capabilities. RGO can alter the activity of matrix metalloproteinases (MMPs), which perform a significant role in cancer development. In particular, it has been shown that RGO has inhibitory effects on MMP-2 and MMP-9, which are known to be upregulated in many cancer types. In general, the use of RGO in the context of cancer treatment has the probability of providing a novel approach to cancer therapy^84^.

Furthermore, MMP2 and 9 overexpression are associated with poor prognosis and metastasizing cancers, as documented by Li et al.^85^. Wang et al.^83^ postulated that MMP2 and MMP9 could augment the invasion and migration of tumor cells by aiding in extracellular matrix degradation. In addition, Li et al.^85^ documented that the RGO downregulates MMP-9 expression, suppressing breast cancer growth and metastasis.

**Figure 7:**
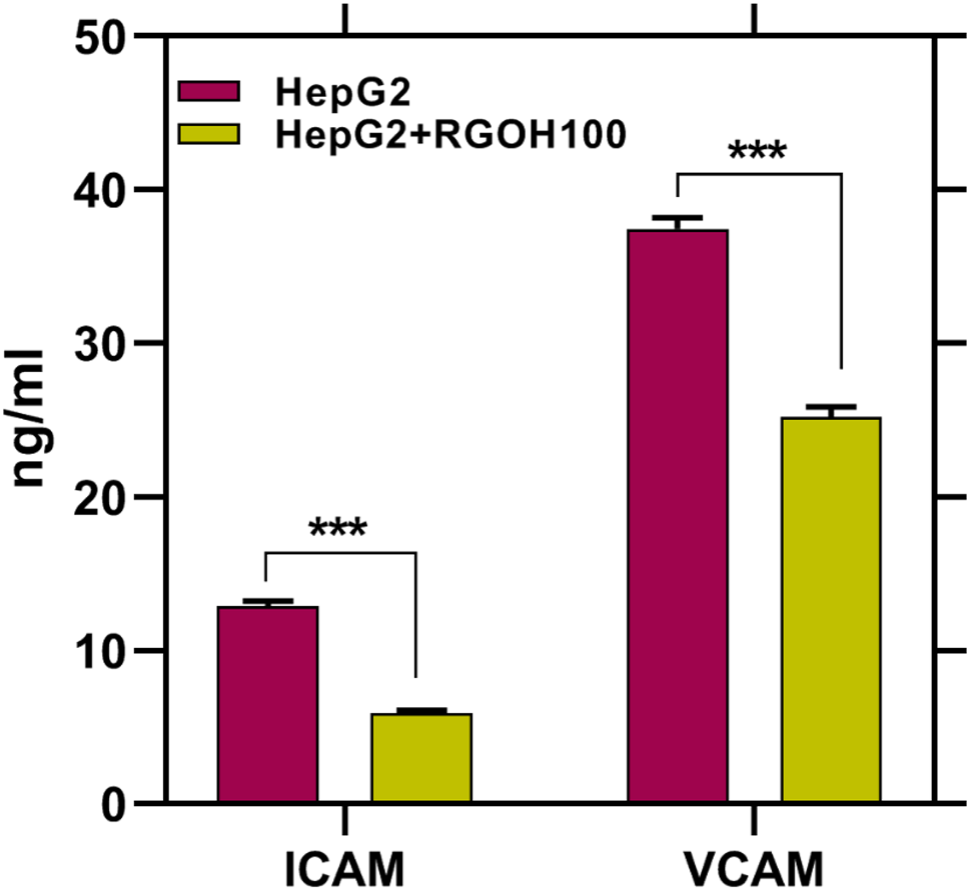
Effect of RGOH100 in cells ICAM and VCAM (ng/mL) in HepG2. Every individual value denotes the mean ± SE (n = 3). Mean values indicate significant differences (***p < 0.001) when compared to HepG2 as the control.

Specific immune cells and regular epithelial and endothelial cells both produce large amounts of cell adhesion molecules, which are transmembrane receptor proteins. These proteins facilitate adhesion between cells and between cells and the extracellular matrix^86^. ICAM-1 and VCAM-1 are adhesion molecules belonging to the Ig superfamily. They could intensify the adhesion between tumor cells and endothelial cells via specific binding to the receptors on the cells, endorsing tumor cell invasion and migration^87^. We looked at the levels of intercellular adhesion molecule (ICAM)-1 and vascular cell adhesion molecule (VCAM)-1 in this study. The results showed that RGOH100 treatment had a significant impact on both proteins in the HCC cell line that represented the tumor group, as shown in Fig. 7^88^. Remarkably, Hosokawa et al.^87^ postulated that ICAM-1 and VCAM-1 are activated by proinflammatory cytokine (IL-1β). Furthermore, Wang et al.^83^, documented that RGO inhibits the expression of ICAM and VCAM metastasis-related proteins^88^.

**Figure 8:**
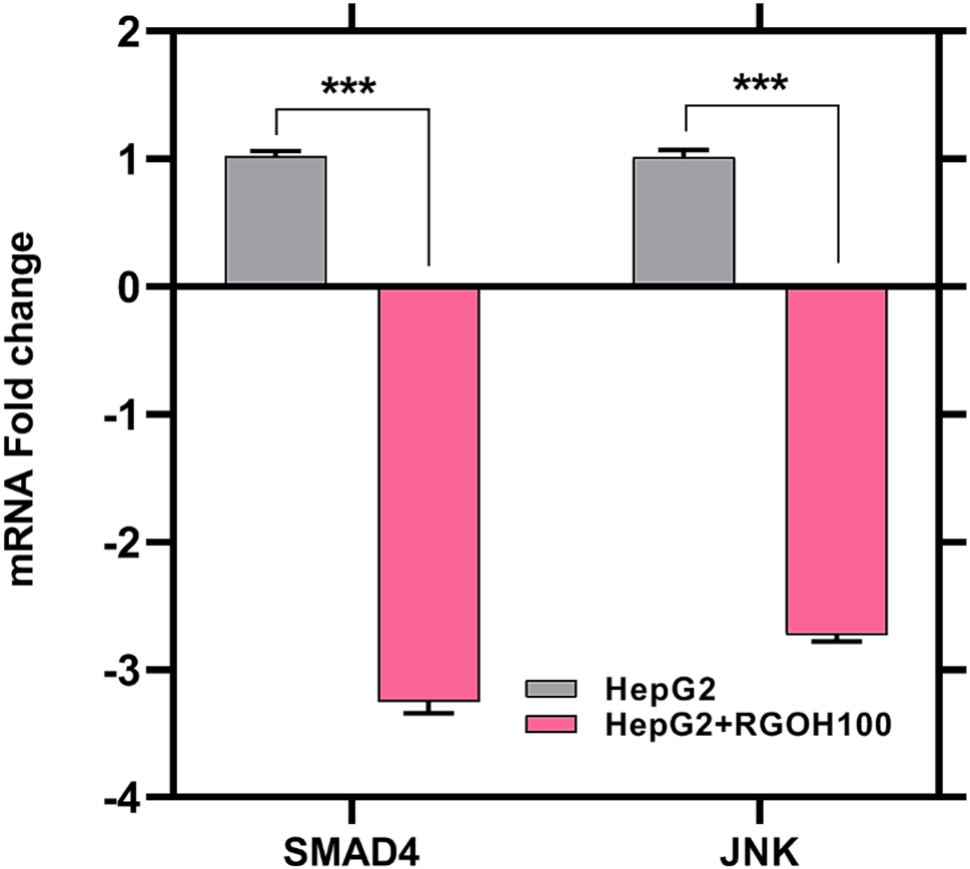
The gene expression of SMAD4 and JNK as ΔCT and fold gene expression in HepG 2 cell line and treatment with RGOH100 (n = 3). The mean ± SE (n = 3). Mean values indicate significant differences (***p < 0.001) when compared to HepG2 as the control.

The Smad4 is categorized as a member of the SMAD protein superfamily and has significant regulatory functions within the TGF-β signaling pathway. The JNK enzyme is part of the mitogen-activated protein kinase (MAPK) family. It is activated in a number of stressful and long-lasting inflammatory conditions that affect various cell types^89^. In this study, the relative expression of JNK decreased after HepG2 cells were treated with RGOH100, as seen in Fig. 8. Additionally, gene expression results displayed a noteworthy down-regulation (p < 0.05) in the group treated with RGOH100 by (-225%) compared to the HCC (HepG2) group. Multiple studies have shown the involvement of JNK in the process of carcinogenesis, namely in digestive system malignancies such as hepatocellular carcinoma (HCC). JNK has been found to facilitate cell proliferation and migration while concurrently impeding apoptosis^90^.

As presented in the Wang et al.^83^ study, RGO has been seen to modulate the SMAD4 and JNK signaling pathways in cancer cells. Through these signaling pathways, RGO in particular downregulates the expression of MMP3 and ICAM. The findings indicate that the suppressive impact of RGO on the production of matrix metalloproteinase 3 (MMP3) and intercellular adhesion molecules (ICAM) is selectively facilitated via the SMAD4 and JNK signaling pathways^83^.

High levels of Smad4 expression have been associated with a poor prognosis following surgery in patients with HCC^33^. In different ways, Smad4 acts as a transcriptional factor and binds to the promoter of TGF-β family receptors, activin receptor type-1B (ACVR1B), and Bone morphogenetic protein receptor type II (BMPR2), and transforming growth factor, beta receptor II (TGFBR2). It then interacts with three coactivators (CREB1, c-JUN, and SP1), which raises the levels of expression even more^91^. Moreover, Smad4 interacts with TGF-β, a molecule that plays a complex function in the progression of cancer. TGF-β acts as a tumor suppressor in the early stages of cancer development while promoting tumor growth in later stages. Additionally, JNK and p38 may phosphorylate the Smad2/3 complex directly or indirectly. This complex then attaches to Smad4 to control downstream gene transcription^90^. So, we can assume that the TGF-/SMAD4 signaling pathway is involved in many cellular processes, such as cell growth, differentiation, apoptosis, migration, and the start and progression of cancer. Increasing SMAD4 levels turn on the TGF-β canonical pathway and boosts the JNK pathway, which causes cancer progression.

## Conclusion

The loading of hesperidin and the successful reduction of GO by γ-rays in the presence of hesperidin were confirmed by different techniques. From FTIR, the removal of oxygen functional groups was confirmed. XRD confirmed that the absence of GO peaks and a broad peak around 24.5° were observed in RGOH25. While the hesperidin peaks were observed upon increasing its concentration above 25 wt.%. It was found that the ID/IG ratio of all RGOH nanocomposites was higher than that of GO. This means that there were more defects and RGOHs were effectively reduced. In addition, RGOH50 has the uppermost ID/IG value, indicating that its graphitic domains are the most plentiful and tiniest compared to those of the other samples. The folded appearance and crumpled morphology of the RGOHs sheets indicate a successful reduction process.

These results from the MTT cytotoxicity test provided evidence that RGOH100 exhibited selective cytotoxic effects on the cancerous cell lines, suggesting its potential use as a therapeutic agent for cancer. Moreover, its anti-inflammatory impact is clarified by inhibiting IL-1B and IL-6 activities and downregulating NF-kB gene expression. Furthermore, the promise of suppressing cancer metastasis is further supported by the antimetastatic effect of reducing ICAM and VCAM activities; furthermore, it downregulates MMP2-9 gene expression. Moreover, RGOH100 might have a role in initiating apoptosis, a desired result in cancer therapy. This effect may be achieved by downregulating bcl2 and the SMAD/JNK signaling pathway while simultaneously upregulating p53 gene expression. In summary, the combined results of these studies underscore the promising application of RGOHs as a nanomaterial in cancer therapy. We primarily attribute this to its demonstrated cytotoxic effects on cancer cell lines, anti-inflammatory characteristics, antimetastatic properties, capacity to induce apoptosis, and ability to inhibit the JNK/SMAD4/MMP2 signaling pathway. However, additional studies are required to investigate the molecular and cellular mechanisms of the antitumor effects of RGOHs on other cancer cell types, as well as in vivo studies. Therefore, it is recommended to conduct more preclinical and post-clinical studies to assess the safety, effectiveness, and biodistribution of these drug leads. Given the limitations of our current work on the P53 and Bcl2 protein expression levels, we will take that into consideration when doing that in future work.

## Data Availability

The datasets used and/or analyzed during the current study available from the corresponding author on reasonable request.
